# Characteristic Study of a Typical Satellite Solar Panel under Mechanical Vibrations

**DOI:** 10.3390/mi15080996

**Published:** 2024-07-31

**Authors:** Xin Shen, Yipeng Wu, Quan Yuan, Junfeng He, Chunhua Zhou, Junfeng Shen

**Affiliations:** 1 State Key Laboratory of Mechanics and Control for Aerospace Structures, Nanjing University of Aeronautics and Astronautics, Nanjing 210016, China; shenxin0413@nuaa.edu.cn (X.S.);; 2 Shanghai Institute of Satellite Engineering, Shanghai 201109, China

**Keywords:** photovoltaic modules, solar panel, mechanical vibration, power generation

## Abstract

As the most common energy source of spacecraft, photovoltaic (PV) power generation has become one of the hottest research fields. During the on-orbit operation of spacecraft, the influence of various uncertain factors and the unbalanced inertial force will make the solar PV wing vibrate and degrade its performance. In this study, we investigated the influence of mechanical vibration on the output characteristics of PV array systems. Specifically, we focused on a three-segment solar panel commonly found on satellites, analyzing both its dynamic response and electrical output characteristics under mechanical vibration using numerical simulation software. The correctness of the simulation model was partly confirmed by experiments. The results showed that the maximum output power of the selected solar panel was reduced by 5.53% and its fill factor exhibited a decline from the original value of 0.8031 to 0.7587, provided that the external load applied on the panel increased to 10 N/m^2^, i.e., the vibration frequency and the maximal deflection angle were 0.3754 Hz and 74.9871°, respectively. These findings highlight a significant decrease in the overall energy conversion efficiency of the solar panel when operating under vibration conditions.

## 1. Introduction

As one of the most significant parts of renewable energy, solar energy has become a hotspot for research worldwide in recent years due to its sustainability and universality. Photovoltaic (PV) systems, which convert the light energy of the sun into electrical energy, represent the most common application of solar power [1]. For example, large-scale solar power plants and rooftop solar panels are now a common sight. And solar power-generation systems are increasingly being mounted on various types of mobile carriers, including cars, satellites, ships, and drones. Additionally, the new concept of floating PV systems on water surfaces is gaining visibility among the public.

Normally, researchers think that the efficiency of photovoltaic power generation is mainly affected by solar radiation and temperature [2,3,4]. For this reason, Cuce et al. [5] experimentally demonstrated that the photogenerated output current, short-circuit current, and other parameters of solar arrays are directly proportional to the light intensity, and the equivalent light intensity is related to the equivalent area of the array perpendicular to the direction of the light. Localized shadows often appear in PV arrays due to interference from the projected coverings of houses, plants, localized dark clouds, surface dust, etc. In this case, the PV system has multiple local maximum power points (MPPs), i.e., its power–voltage (P-V) curve exhibits a multi-peak characteristic [6,7,8]. Abdulmawjood et al. [9] analyzed the impact of different shading patterns on the P-V characteristics through performing a set of simulations with different array configurations. It was found through experiments that the power dropped significantly when the shading increased. Under localized shadows, PV arrays also produce hotspot effects, resulting in multi-peak-output P-V curves, at which time the conventional maximum power point tracking (MPPT) is prone to local extreme points and failure [10]. In addition, as the main factor affecting the performance of photovoltaic power generation, solar radiation’s own randomness and cyclical changes will make the power of photovoltaic power generation intermittent and fluctuating [11,12].

In consideration of the application environment of PV systems, it is easy to find that mechanical vibration always exists and may also have an influence. However, there has not been a lot of public interest in this. Although not particularly emphasized in engineering, some scholars have conducted research in response to such questions. Domestic large-scale photovoltaic power plants are mainly distributed in the northwest and other light-rich regions of China, where the windy climate also brings a negative impact on the efficient operation of photovoltaic power-generation systems. With the help of experiments, scholars have demonstrated that wind-induced vibration leads to oscillations of output current [13,14], and they have inferred that, in the low-frequency range, vibration-induced current transients and oscillations in the output of PV modules are the main sources of distortion. Vibrations from the carrier motion itself or from the carrier engine operation can also cause the oscillation of the PV module mounted on the carrier surface. Studies by Zhang [15] investigated PV components on a train, proving that the carrier vibration will lead to changes in the output characteristics, which will in turn lead to the failure of the original MPPT control. Vidović et al. [16] focused on ocean floating solar power-generation devices, and combined a static solar radiation energy calculation model with the dynamic swing of the float angle to determine that the radiation energy received by the float and photovoltaic cells under the influence of waves was 87.5% of the solar radiation energy received by photovoltaic cells fixedly installed at the optimal receiving angle under the same conditions.

Although mechanical vibration energy can be collected and converted into electrical energy [17,18], the power-generation reduction in PV systems does exist and strongly complicates the MPPT algorithm under vibration conditions. Moreover, as the sole power source for most satellites, solar arrays are usually large outreach structures, and their size is increasing due to their growing number of tasks and power requirements [19]. During on-orbit operation, the influence of various uncertain factors in the space environment and the unbalanced inertial force can easily cause solar arrays to vibrate and degrade their performance [20]. Therefore, to gain a deeper understanding of the impact of mechanical vibration on the output characteristics of PV systems and to meet the engineering application needs, this study takes a classical three-segment satellite solar panel as the research object and mainly calculates its dynamic response and electrical performance under mechanical vibration, and the effects are systematically discussed. This study provides new insights for future research on structural vibration suppression and the MPPT algorithm in satellite solar panels, and the research process is also applicable to the analysis of ground-based PV systems subject to vibration, especially in times of dramatic climate change.

This paper is structured as follows: Section 2 outlines the structural dynamics and photovoltaic power modeling of a typical satellite solar panel. Section 3 details the characterization of the solar panel under specific conditions. Finally, the conclusions are presented in Section 4.

## 2. Structural Dynamics and Photovoltaic Power Model of a Typical Solar Panel

### 2.1. Structural Dynamics Model of a Typical Solar Panel

**Figure 1:**
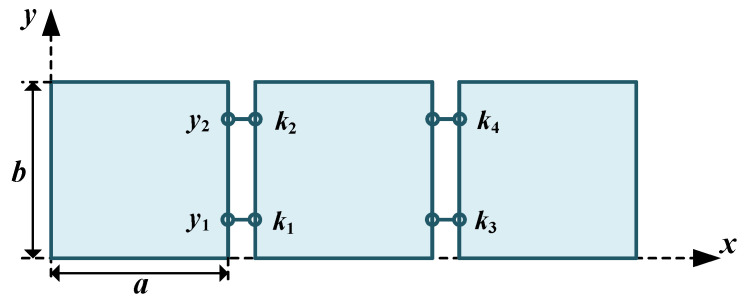
The schematic solar panel structure with three sub-plates.

A satellite solar panel is a large-span, highly flexible, external extension structural system, but the individual rectangular panels that constitute its basic units can be well described using a linear model [21]. Therefore, in this paper, the three-segment solar panel is represented by a typical model of three elastic rectangular thin plates connected with micro-torsion springs. As shown in Figure 1, Plate-1 was fixedly attached to the spacecraft body and connected to Plate-2 by two torsion springs *k*_1_ and *k*_2_, and Plate-2 was connected to Plate-3 by torsion springs *k*_3_ and *k*_4_. In the figure, *a* and *b* represent the side lengths of a single rectangular plate in the *x* and *y* directions, respectively, while *y*_1_ and *y*_2_ represent the connection positions of torsion springs *k*_1_, *k*_3_ and *k*_2_, *k*_4_ in the *y* direction, respectively. The follow-up studies were based on the above model with the following assumptions [21,22]:

1. The solar array consisted of three rectangular plates connected by torsion springs, and the substructures, such as rectangular plates and springs, can be described by a linear model. This means that the deformation of the plates and the torsion springs was within the linear elasticity range, and the geometric and material nonlinearity of the structure’s deformation was not considered.
2. The rigid-body motion of the spacecraft flight was fixed, and only the elastic vibration of the panel is discussed. The first plate was fixed to the spacecraft body, and the reference coordinate system is shown in Figure 1.
3. The two plates were connected by two torsion springs, which had rotational freedom only around the y-axis. The torsion springs and joints were small, and their structures ensured the effective transmission of torque, shear force, and axial force.
4. The panel formed a stable system when it was completely unfolded, meaning that the rectangular plates and the connecting torsion springs were well fixed.
5. The geometry and mass of the torsion spring joints were neglected. The axial and shear deformation of the torsion springs was not considered; only the change in the torsion angle was considered.
6. The longitudinal stiffness of the solar panel was very large, and the waving vibration was negligible.

#### 2.1.1. Deflection Model of the Solar Panel

The key to determining the dynamic response of the structure is to determine the form of deflection and the deflection value of the structure. Still referring to Figure 1, it is easy to see that Plate-1 was fixed on one side, free on both sides, and connected to torsion springs on one side; two sides of Plate-2 were free and the other two were connected to torsion springs; and three sides of Plate-3 were free and the other was connected to torsion springs. The three plates represent all three possible cases with different boundary conditions in multi-plate connected structures.

**Figure 2:**
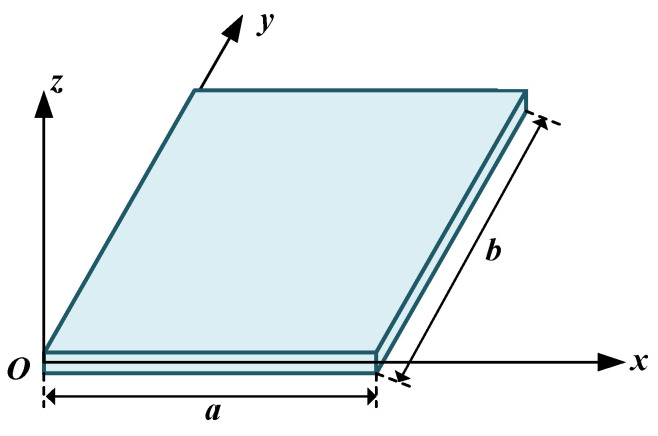
The thin plate and the corresponding coordinates.

As shown in Figure 2, the boundary conditions for the solidly supported edges of Plate-1 were displacement boundary conditions. *w*_1_(*x*,*y*), *w*_2_(*x*,*y*), and *w*_3_(*x*,*y*) represent the deflections at the ends of the three thin plates, and approximately satisfy the bending moment boundary conditions at the connected edges. According to the different boundary conditions, the deflection functions adopted the form of the separation of variables *w*_i_(*x*,*y*) = *A*_i_(*y*)*B*_i_(*x*), and the deflection function for each plate is listed below [21].

Plate-1:

(1) $$\begin{array}{ll} w_{1}(x,y)= & (c_{1}+c_{2}\overset{n}{\underset{i=1}{\sum }}\frac{b^{2}}{i^{2}\pi^{2}}\cos\frac{i\pi y_{1}}{b}\cos\frac{i\pi y}{b}+c_{3}\overset{n}{\underset{i=1}{\sum }}\frac{b^{2}}{i^{2}\pi^{2}}\cos\frac{i\pi y_{2}}{b}\cos\frac{i\pi y}{b}\\ & +c_{4}y^{2}+c_{5}y)\times (1-\cos\frac{\pi x}{2a}+c_{6}x^{2}) \end{array}$$

Plate-2:

(2) $$\begin{array}{ll} w_{2}(x,y)= & (c_{7}+c_{8}\overset{n}{\underset{i=1}{\sum }}\frac{b^{2}}{i^{2}\pi^{2}}\cos\frac{i\pi y_{1}}{b}\cos\frac{i\pi y}{b}+c_{9}\overset{n}{\underset{i=1}{\sum }}\frac{b^{2}}{i^{2}\pi^{2}}\cos\frac{i\pi y_{2}}{b}\cos\frac{i\pi y}{b}\\ & +c_{10}y^{2}+c_{11}y)\times \left[1+c_{12}(x-a)+c_{13}(x-a{)}^{2}+c_{14}(x-a{)}^{3}\right] \end{array}$$

Plate-3:

(3) $$\begin{array}{ll} w_{3}(x,y)= & (c_{15}+c_{16}\overset{n}{\underset{i=1}{\sum }}\frac{b^{2}}{i^{2}\pi^{2}}\cos\frac{i\pi y_{1}}{b}\cos\frac{i\pi y}{b}+c_{17}\overset{n}{\underset{i=1}{\sum }}\frac{b^{2}}{i^{2}\pi^{2}}\cos\frac{i\pi y_{2}}{b}\cos\frac{i\pi y}{b}\\ & +c_{18}y^{2}+c_{19}y)\times \left[1+c_{20}(x-2a)+c_{21}(x-2a{)}^{2}\right] \end{array}$$

where *c*_m_ (m = 1, 2, …, 21) in the functions are coefficients to be solved, and *n* is the truncation term.

Based on the energy equation of the system and the constraint that the torsion spring has only one rotational degree of freedom, the coefficient *c*_m_ in the selected deflection function was calculated using the Ritz method. It was assumed that the solar cell wing was combined with three square plates of the same material, which was isotropic and orthotropic. The three plates were subjected to a uniform load *q*. The connection positions were taken as *y*_1_ = 0.25*b* and *y*_2_ = 0.75*b*, the side lengths of a square plate as *a* = *b* = 1 m, the density as *ρ* = 1700 kg/m^3^, the modulus of elasticity as *E* = 1.5 × 10^11^ Pa, the modulus of stiffness as *D*_2_ = 0.5*D*_1_ and *D*_3_ = 1.215*D*_1_, and Poisson’s ratio as *μ*_1_ = 2*μ*_2_ = 0.3. The four micro-torsion springs had the same coefficient of elasticity, i.e., *k*_1_ = *k*_2_ = *k*_3_ = *k*_4_ = 300*D*_1_, and the truncation term *n* was chosen to be 15. Then, an expression for the deflection of the solar panel under an external load can be obtained.

Plate-1:

(4) $$\begin{array}{ll} w_{1}(x,y)= & \frac{q}{D_{1}}(0.791349+0.0544\overset{15}{\underset{i=1}{\sum }}\frac{1}{4i^{2}\pi^{2}}\cos\frac{i\pi }{2}\cos2i\pi y-0.132467y^{2}\\ & +0.132467y)\times (1-\cos\frac{\pi x}{2a}+1.196593x^{2}) \end{array}$$

Plate-2:

(5) $$\begin{array}{cc} w_{2}(x,y)= & \frac{q}{D_{1}}(1.78621-0.012\overset{15}{\underset{i=1}{\sum }}\frac{1}{4i^{2}\pi^{2}}\cos\frac{i\pi }{2}\cos2i\pi y-0.042y^{2}+0.042y)\\ & \times \left[1+1.806462\;(x-a)+0.544749\;(x-a{)}^{2}-0.159476(x-a{)}^{3}\right] \end{array}$$

Plate-3:

(6) $$\begin{array}{ll} w_{3}(x,y)= & \frac{q}{D_{1}}(5.723103-0.00431\overset{15}{\underset{i=1}{\sum }}\frac{1}{4i^{2}\pi^{2}}\cos\frac{i\pi }{2}\cos2i\pi y+0.015087y^{2}\\ & +0.015087y)\times \left[1+0.8363989\;(x-2a)+0.020779\;(x-2a{)}^{2}\right] \end{array}$$

**Figure 3:**
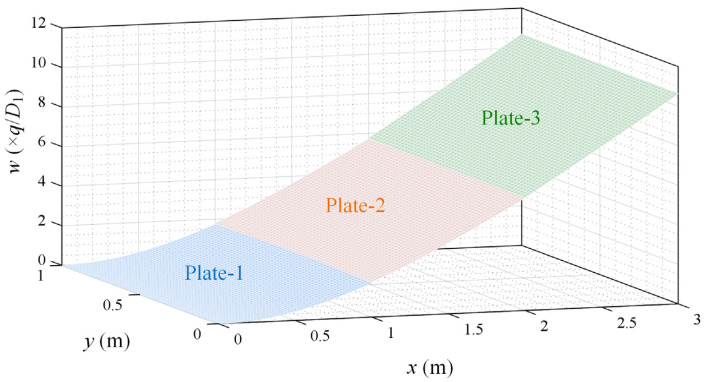
Deflection of the three-plate structure under a uniform load *q*.

The result in Figure 3 was obtained by MATLAB programming. It was found that, under a uniform load *q*, the deflection of the panel was symmetrical about *y* = 0.5*b* and the deflection reached its maximum at *y* = 0.5*b*. Overall, the difference in the deflection of the solar panels in the *y* direction was quite small. Therefore, the deflection of the three-plate structure at *y* = 0.5*b* was taken as the object of study to simplify the plate structure.

**Figure 4:**
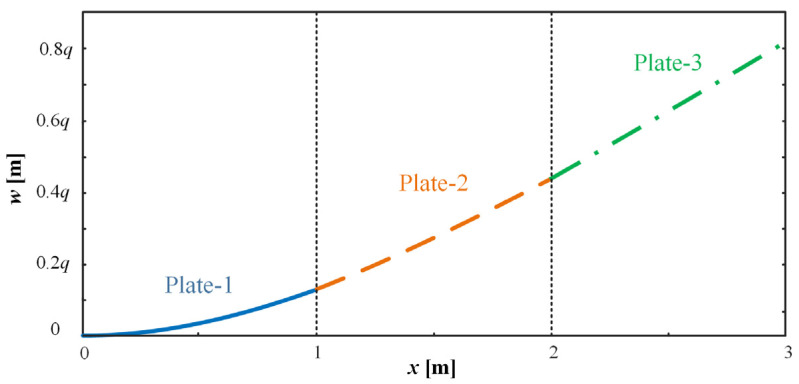
Deflection curves for three-plate structures.

The deflection of each plate was at the maximum at its end. To further simplify the study and to facilitate the application of the results to subsequent studies, the average deflection of each plate was calculated and the rotation angle of each plate was derived from the average deflection. When the uniform load *q* was 1 N/m^2^, 3 N/m^2^, 5 N/m^2^, or 10 N/m^2^, respectively, the deflections at each of the three plates were calculated, as shown in Figure 4. The resulting angles of rotation are listed in Table 1, at the end of this subsection.

#### 2.1.2. First-Order Resonant Frequency of the Solar Panel

Since the solar panel was characterized by a large span, a light mass, and large flexure, it was easy to produce violent and long-lasting vibrations after receiving small perturbations, and these low-frequency vibrations were mainly manifested as transverse bending and vibrations perpendicular to the panel surface.

In the selected model, the stiffness coefficient of the torsion springs between the plates was much larger than the bending stiffness coefficient of the plates. In analyzing the resonant frequency, when the panel was unfolded, if the stiffness of the locking structure was large enough, the solar panel composed of multiple plates could be approximated as a single flexural rectangular plate. Therefore, in this subsection, the three-plate model was simplified to a single plate for the analysis. The side length *b* in the *y* direction equaled 1m and the side length 3*a* in the *x* direction equaled 3m. The density of the material, the form of the external force applied to the panel, and the magnitude of the external force (uniformly distributed load *q*) were the same as in the previous subsection.

The vibration mode function of the solar panel was chosen as follows:

(7) $$W\left(x,y\right)=\left[c_{1}+c_{2}\sin\frac{\left(2k-1\right)\pi y}{2a\sqrt{2-\mu }}+c_{3}\cos\frac{\left(2k-1\right)\pi y}{2a\sqrt{2-\mu }}\right]\times \left[1-\cos\frac{\left(2k-1\right)\pi x}{2a}\right]$$

If the order of the vibration mode is determined as *k* = 1, the first-order mode function of the panel can be determined using the Ritz method. Subsequently, by applying the law of conservation of energy to the system, a series of frequency values can be calculated, and the smallest positive real root can be identified as the first-order resonant frequency *ω* of the panel. According to the above method, the first mode frequency of the solar panel was obtained as 0.3754 Hz with the help of the numerical calculation software. So far, the parameters and vibration characteristics of the three-segment solar panel are listed in Table 1.

### 2.2. Photovoltaic Power-Generation Model of a Typical Solar Panel

**Figure 5:**
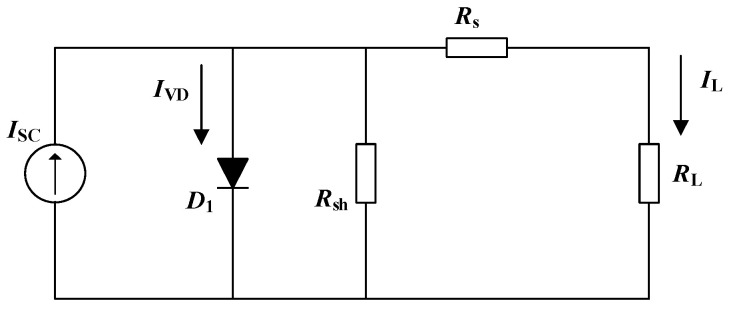
Equivalent circuit of photovoltaic cell.

A photovoltaic module consists of solar cells connected in series and in parallel. A solar cell can usually be represented as a current source and a diode in parallel [23,24], as shown in Figure 5. 

And there is a practical mathematical model for engineering use, as follows [25]:

(8) $$\begin{array}{c} I_{L}=I_{sc}\left\{1-C_{1}\left[\left(e^{\frac{V_{L}}{C_{2}V_{oc}}}-1\right)-1\right]\right\} \end{array}$$

(9) $$\begin{array}{c} C_{1}=\left(1-\frac{I_{m}}{I_{sc}}\right)e^{-\frac{V_{m}}{C_{2}V_{oc}}} \end{array}$$

(10) $$\begin{array}{c} C_{2}=\left(\frac{V_{m}}{V_{oc}}-1\right){\left[\ln\left(1-\frac{I_{m}}{I_{sc}}\right)\right]}^{-1} \end{array}$$

Equation (8) depicts the characteristic curve of a solar cell for a standard irradiance of *S*_ref_ = 1000 W/m^2^ and a standard temperature of *T*_ref_ = 25 °C. However, the coefficients *C*_1_, *C*_2_, *V*_oc_, and *I*_sc_ will vary due to the influence of the external sunlight intensity and the ambient temperature. When the irradiance and reference temperature change, the formula becomes inapplicable and needs to be adjusted to describe the new curve. The improving method is to derive *I*_sc_′, *V*_oc_′, *I*_m_′, and *V*_m_′ under general working conditions (irradiance *S* and temperature *T*) from the *I*_sc_, *V*_oc_, *I*_m_, and *V*_m_ under the standard sunlight intensity *S*_ref_ and the standard temperature *T*_ref_. Then, Equation (8) can still be utilized to perform engineering calculations of the output characteristics under non-standard working conditions.

First, the temperature difference Δ*T* and the relative irradiance difference Δ*S* between the general and standard working conditions were calculated as follows:

(11) $$\Delta T=T-T_{ref}$$

(12) $$\Delta S=\frac{S}{S_{ref}}-1$$

Then, *I*_sc_′, *V*_oc_′, *I*_m_′, and *V*_m_′ under general working conditions were calculated using the following formulae.

(13) $$I_{sc}^{\prime }=I_{sc}\frac{S}{S_{ref}}(1+g\Delta T)$$

(14) $$V_{oc}^{\prime }=V_{oc}(1-k\Delta T)\ln(e+h\Delta S)$$

(15) $$\begin{array}{c} I_{m}^{\prime }=I_{m}\frac{S}{S_{ref}}(1+g\Delta T) \end{array}$$

(16) $$\begin{array}{c} V_{m}^{\prime }=V_{m}(1-k\Delta T)\ln(e+h\Delta S) \end{array}$$

The projection process assumed that the basic shape of the output curve was unchanged; the typical values of coefficients g, h, and k were 0.0025/°C, 0.5/(W/m^2^), and 0.00288/°C, respectively.

By replacing *I*_sc_, *V*_oc_, *I*_m_, and *V*_m_ under the standard conditions with the obtained *I*_sc_′, *V*_oc_′, *I*_m_′, and *V*_m_′ under the new conditions, *C*_1_′ and *C*_2_′ under the new conditions can be obtained with Equations (9) and (10), thus solving the problem of calculating the output characteristics under any irradiance and temperature with Equation (8).

## 3. Structural Dynamics and Photovoltaic Power Modeling of a Typical Solar Panel

### 3.1. Parameter Validation of Photovoltaic Power-Generation Model under Dynamic Conditions

In order to obtain the output characteristic curves of the solar panel and to ascertain the influence of external environmental factors on its output characteristics, this section utilized MATLAB/Simulink(R2020a) to conduct an output simulation of the solar cell. This was based on the research content of the previous sections, and the correctness of the model was verified experimentally.

The data for the selected solar panel were provided by the manufacturer, and the solar panel’s parameters under standard conditions are shown in Table 2.

#### 3.1.1. Simulation of Photovoltaic Power-Generation Model

**Figure 6:**
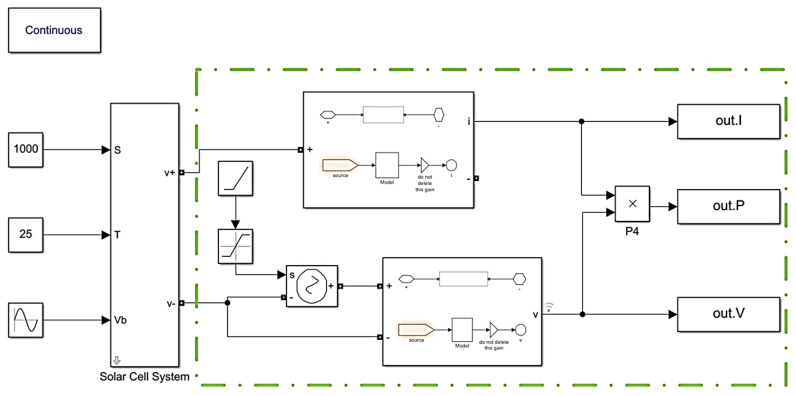
Single solar panel simulation.

Figure 6 shows the simulation model for a single solar panel. Inside the dashed box lies the external measurement circuit used to capture the panel’s output current, voltage, and power. The input ‘S’ in the model represents the solar irradiance, measured in units of W/m^2^. The input ‘T’ signifies the ambient temperature, measured in °C. And the input ‘Vb’ denotes the vibration, which can be adjusted based on different vibration characteristics, including the vibration form, frequency (Hz), and vibration angle (rad). It is important to note that ‘Vb’ is in the form of an angle.

It is worth mentioning that, in past research, it was often tacitly assumed that the angle of incidence of sunlight and the output current conform to the cosine theorem. In practice, however, when the angle of incidence exceeds 55°, the value of the output current gradually deviates from the cosine value, and when it exceeds about 85°, there is no output power from the solar cell, although theoretically, there should still be 7.5%. The output power curve of a real solar cell as a function of the angle of incidence of the sun is known as the Kelly cosine. For the sake of rigor, this paper characterizes the Kelly cosine relationship between the output current and the angle of incidence *θ* based on an empirical formula proposed in the literature [26]:

(17) $$\begin{array}{c} I_{L}=\max\left(I_{L0}\cos\theta -a\cdot u\left(|\theta |-\theta_{th}\right)\cdot \left(|\theta |-\theta_{th}\right),0\right) \end{array}$$

The threshold angle *θ*th was set to 55°, and when the angle of incidence was less than the threshold, the experimental data conformed to the standard cosine law. When the angle of incidence exceeded the threshold, the experimental data could be more accurately modelled using the empirical Equation (17).

**Figure 7:**
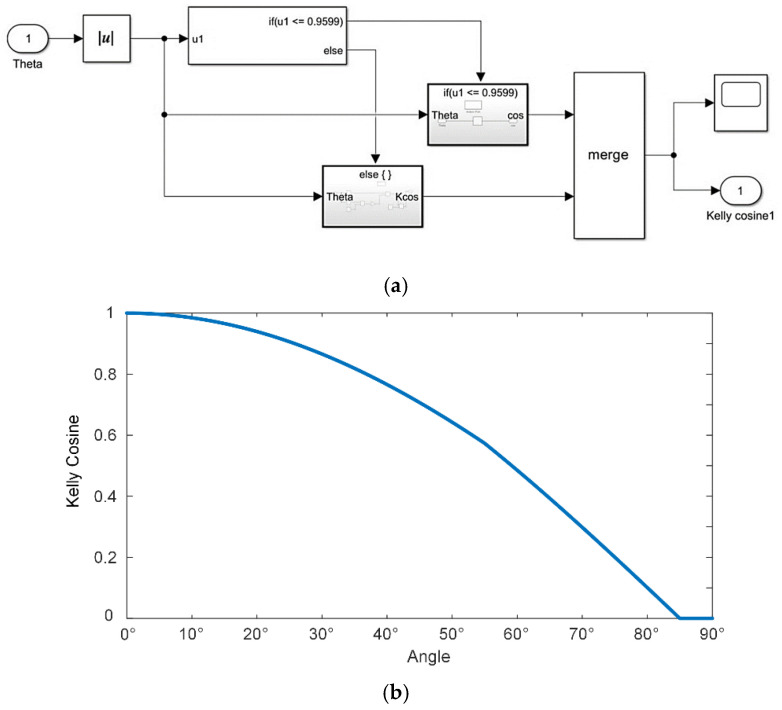
Kelly cosine applied to PV systems and its simulation result. (**a**) Kelly cosine simulation. (**b**) Kelly cosine value as a function of the angle.

The model was built in Simulink according to the empirical formula, and the output results were basically consistent with the theoretical data, as shown in Figure 7. 

To delve into the impact of generating multiple localized poles on the maximum output power of the solar panel under dynamic conditions, two sets of simulations were conducted. These analyses aimed to compare and analyze the effects on the MPP of two significant parameters influencing mechanical vibrations: the frequency and the vibration angle. This investigation was crucial due to the multi-polar characteristics of the solar panel’s output power under dynamic conditions.

**Figure 8:**
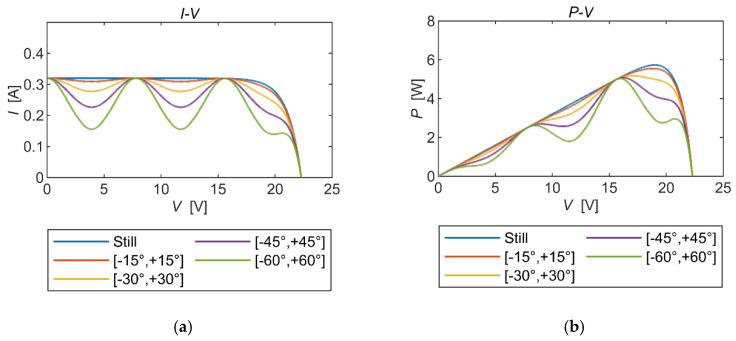
PV properties of the same vibration frequency with different vibration angles. (**a**) The *I*-*V* curves. (**b**) The *P*-*V* curves.

From Figure 8, it is clear that the same number of local extremes occurred at the same frequency of mechanical vibration. As the mechanical vibration angle increased, the output power showed a sudden rise-and-fall characteristic with voltage escalation, and this tendency became more pronounced with a higher vibration angle. Moreover, the maximum power output showed a decreasing trend as the vibration angle increased.

**Figure 9:**
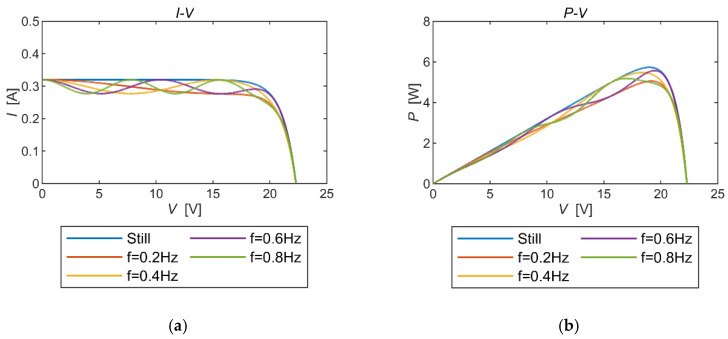
PV properties of the same vibration angle with different vibration frequencies. (**a**) The *I*-*V* curves. (**b**) The *P*-*V* curves.

In Figure 9, it was observed that, for the same vibration angle, the number of local extreme points increased with an increase in the vibration frequency. However, the output power curve consistently remained near the static output power curve. The maximum output power in this case showed fluctuating changes. Assuming that the instrument operates at the MPP, it is generally expected that the power loss will decrease as the frequency increases. Nevertheless, this pattern did not hold true in the low-frequency range.

#### 3.1.2. Validation of Photovoltaic Power-Generation Model

**Figure 10:**
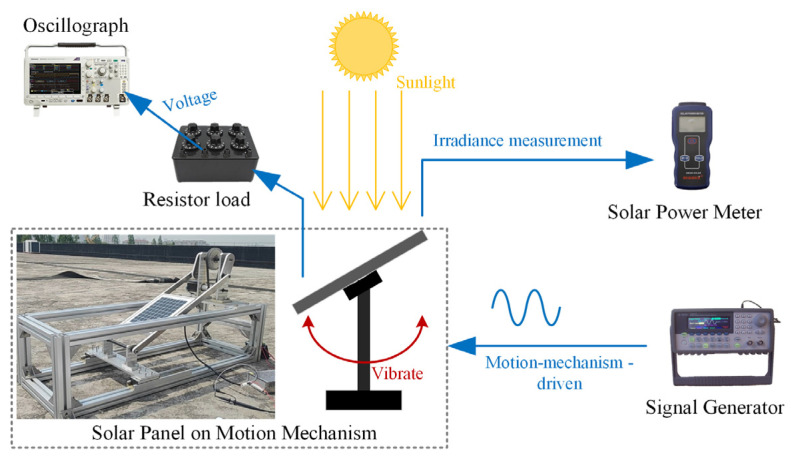
Schematic diagram of the experimental setup.

A solar panel photovoltaic characterization experiment was carried out to verify the correctness of the simulation model. The equipment mainly included an oscilloscope, a resistance box, a solar power meter, a signal generator, and a solar panel. Figure 10 illustrates a schematic diagram of the connection of the experimental setup.

**Figure 11:**
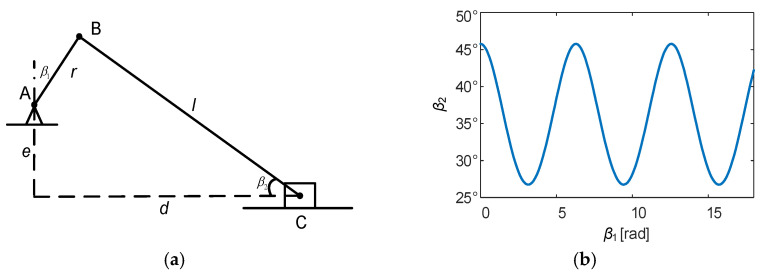
Mechanical sketch and calculations of the motion mechanism. (**a**) Mechanical sketch. (**b**) Calculations of the angle between the solar panel and the ground.

Additionally, a motion mechanism was employed to simulate the vibration of the solar panel and its mechanical sketch is presented in Figure 11. The angles *β*_1_ and *β*_2_ satisfy the following system of equations:

(18) $$\left\{\begin{array}{l} \begin{array}{c} {\left(d-r\sin\beta_{1}\right)}^{2}+{\left(e+r\cos\beta_{1}\right)}^{2}=l^{2} \end{array}\\ \begin{array}{c} cos\beta_{2}=\frac{d-r\sin\beta_{1}}{l} \end{array} \end{array}\right.$$

Taking *e* as 350 mm, *r* as 80 mm, and *l* as 600 mm, the relationship between the angles *β*_1_ and *β*_2_ can be represented as follows:

(19) $$\begin{array}{c} \beta_{2}=8sin\beta_{1}+{\left(64{\left(\sin\beta_{1}\right)}^{2}-560\cos\beta_{1}+2311\right)}^{2} \end{array}$$

According to the calculated results, the variation interval of the angle between the solar panel and the ground was [26.7440°, 45.7800°]. In addition to this, the actual solar incidence angle, which was used in the simulation model, needed to be calculated based on the local latitude of the experiment, the solar time angle, the solar declination angle, and the panel azimuth angle [27,28].

**Figure 12:**
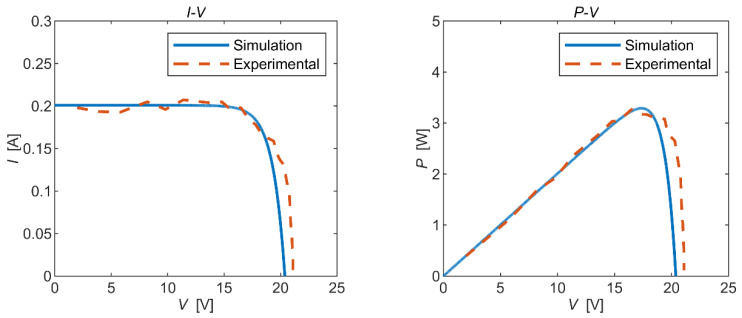
Static experimental results.

**Figure 13:**
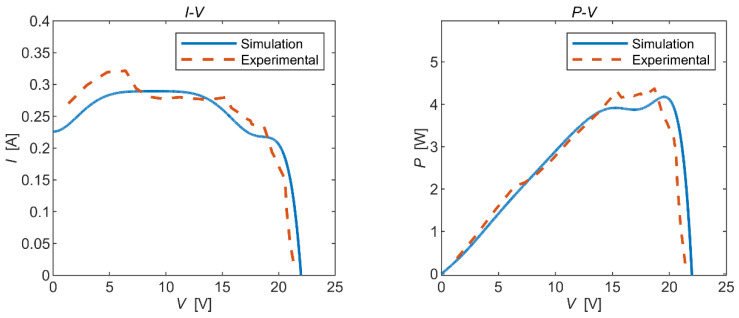
Dynamic experimental results.

Static and dynamic experiments were conducted, respectively, and the experimental results were compared with the simulation results as follows:

It can be seen from Figure 12 and Figure 13 that the trends in the experimental curves closely aligned with the simulation curves, providing evidence of the feasibility of the simulation model. However, there were some discrepancies between certain experimental data points and the simulation results, indicating a degree of deviation. Upon analysis, potential sources of error in the experimental results included the following: (1) Natural sunlight is unstable and the irradiation is in real-time fluctuation; hence, there existed a large error when reading the output voltage under a specific irradiance. (2) In order to read the test data under a specific irradiance, the time span of the experiment was large, and the solar time angle changed slowly during the process, while the solar incident angle was calculated at a certain moment in the experimental time, which affected the accuracy of the data results. (3) Compared with the theoretical results, the open-circuit voltage obtained from the static experiments was large because the internal resistance of the solar panel varied with changes in the light intensity, cell temperature, and output voltage. In addition, the factory parameters of solar panels provided by the manufacturer are the data for the same batch of panels, and the actual parameters of the panels may not be completely consistent with the standard parameters.

### 3.2. Power-Generation Characterization of a Typical Solar Panel under Dynamic Conditions

From the above experimental results, the simulation model was constructed with a certain correctness and feasibility. In this subsection, the model of this single solar panel was utilized to form a solar cell array in series, which was used to simulate the output of a solar panel with a three-plate structure under vibration conditions.

When solar panels are connected in series, the vibration condition of each plate is generally different. In general, the amplitude of the plate solidly connected to the spacecraft body is the smallest, and the plates farther away from the body have larger vibration amplitudes. From the output characteristics of a single panel, it can be hypothesized that, under vibration conditions, the output power of the solar array when multiple panels are connected in series also exhibits multipolar characteristics. The vibration frequency of the solar cell wing and the average rotation angle of each plate under different uniform loads were obtained through the kinetic calculation of the solar cell wing in Section 2. By inputting each set of data into the simulation model separately, the output characteristics of the solar cell wing under specific vibration conditions were obtained. 

**Figure 14:**
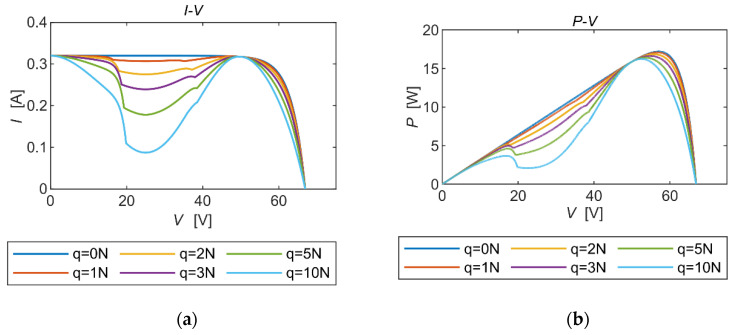
Power-generation characterization. (**a**) The *I*-*V* curves. (**b**) The *P*-*V* curves.

**Figure 15:**
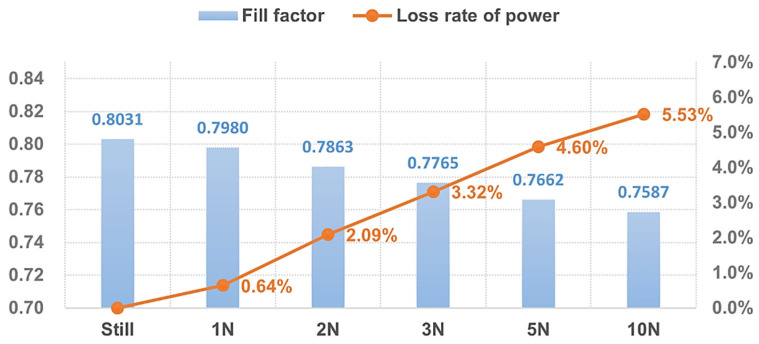
Loss of output power of the solar panel under a uniform load.

According to Figure 14, there were few cases of localized extreme points in the *P*-*V* curve when the external uniform load was small. As the external load increased, the vibration of the solar panel became more intense, the indentation of the curve was more pronounced, and its loss of output power increased. When the vibration frequency and the maximal deflection angle were 0.3754 Hz and 74.9871°, the maximum output power of the selected solar PV wing was reduced by 5.53%, which is quite considerable. The magnitude of maximum power reduction seemed to decrease with an increasing load, as shown in Figure 15.

It is noteworthy that the fill factor (FF) is an important performance indicator when analyzing the performance of solar cells. It is the ratio of the maximum output power of the battery to the product of the maximum value of the short-circuit current and open-circuit voltage, as shown in Equation (20). The FF reflects the ability and efficiency of a solar cell to use light energy during operation. It is a key parameter that is used to measure the performance of solar cells, with a typical range of 0.5 to 0.9, and the closer it is to 1, the better the performance. Still referring to Figure 15, obviously, as the external load increased to 10 N/m^2^, the FF of the solar panel exhibited a decline from its original value of 0.8031 to 0.7587, which indicates that the overall energy conversion efficiency of the solar panel decreased significantly under vibration conditions.

(20) $$FF=\frac{P_{M}}{V_{OC}\times I_{SC}}$$

## 4. Conclusions

This paper analyzed the mechanical oscillation characteristics of the solar panel of a spacecraft, which is a typical application scenario of PV modules. By integrating a solar cell simulation model, this study established the output behavior of solar arrays under mechanical vibration conditions and experimentally verified its correctness using a PV testing platform. While the impact of mechanical vibration on the output characteristics of the solar panel may seem like a minor detail, it holds significant importance for spacecraft operations. And as solar arrays become even larger, the seemingly acceptable power loss on each solar panel will add up to a huge waste.

By examining the power-generation quality of solar arrays and evaluating the output voltage, power, and other parameters affected by mechanical vibration, we can identify performance improvement indices after the stabilization of the structure. This offers new insights for optimizing spacecraft structure and power supply systems, ultimately forming an evaluation model for enhancing the power supply efficiency and providing guidelines for structural optimization.

However, it is important to note that this study only considered the vibration characteristics under a uniform load in the mechanical analysis of a solar cell wing. In actual scenarios, spacecraft motion involves attitude adjustments, re-orbiting motion, and the extension and retraction of the battery wing, resulting in a highly complex and variable force situation. Further research and improvement efforts are required to comprehensively address these challenges.

## Figures and Tables

**Table 1:** Structural dynamics parameters of solar panel with 3 plates.

		Plate-1	Plate-2	Plate-3
Rectangular plate size: *a* × *b*		1 × 1 m × m		
Density: *ρ*		1700 kg/m^3^		
Elastic modulus: *E*		1.5 × 10^11^ Pa		
Poisson’s ratio		*μ*_1_= 0.3; *μ*_2_ = 0.15		
Torsion spring positions		*y*_1_ = 0.25 m; *y*_2_ = 0.75 m		
First-order resonant frequency: *f*		0.3754 Hz		
Rotation angle	*q* = 1 N/m^2^	7.1830°	16.4648°	20.4489°
	*q* = 2 N/m^2^	14.1470°	30.5870°	36.7132°
	*q* = 3 N/m^2^	20.7107°	41.5614°	48.2042°
	*q* = 5 N/m^2^	32.2165°	55.9132°	61.7917°
	*q* = 10 N/m^2^	51.5687°	71.3064°	74.9871°

**Table 2:** Solar panel parameters.

Parameters	Names	Value
Short-circuit current	*I_sc_*	0.32 A
Open-circuit voltage	*V_oc_*	22.3 V
Maximum power point current	*T_m_*	0.28 A
Maximum power point voltage	*V_m_*	17.90 V

## Data Availability

Data is contained within the article.
